# Deep Phenotyping of Urinary Leukocytes by Mass Cytometry Reveals a Leukocyte Signature for Early and Non-Invasive Prediction of Response to Treatment in Active Lupus Nephritis

**DOI:** 10.3389/fimmu.2020.00256

**Published:** 2020-03-24

**Authors:** Martina Bertolo, Sabine Baumgart, Pawel Durek, Anette Peddinghaus, Henrik Mei, Thomas Rose, Philipp Enghard, Andreas Grützkau

**Affiliations:** ^1^Department of Nephrology, Charité Universitätsmedizin Berlin, Berlin, Germany; ^2^Deutsches Rheuma-Forschungszentrum Berlin (DRFZ), a Leibniz-Institute, Berlin, Germany; ^3^Department of Rheumatology and Clinical Immunology, Charité Universitätsmedizin Berlin, Berlin, Germany

**Keywords:** systemic lupus – erythematosus, lupus nephritis, lupus nephritis biomarker, mass cytometry, urinary leukocytes

## Abstract

Non-invasive biomarkers are necessary for diagnosis and monitoring disease activity in lupus nephritis (LN) to circumvent risks and limitations of renal biopsies. To identify new non-invasive cellular biomarkers in the urine sediment of LN patients, which may reflect kidney inflammation and can be used to predict treatment outcome, we performed in-depth urinary immune cell profiling by mass cytometry. We established a mass cytometric workflow to comparatively analyze the cellular composition of urine and peripheral blood (PB) in 13 patients with systemic lupus erythematosus (SLE) with active, biopsy-proven proliferative LN. Clinical and laboratory data were collected at the time of sampling and 6 months after induction of therapy in order to evaluate the clinical response of each patient. Six patients with different acute inflammatory renal diseases were included as comparison group. Leukocyte phenotypes and composition differed significantly between urine and paired PB samples. In urine, neutrophils and monocytes/macrophages were identified as the most prominent cell populations comprising together about 30%–83% of nucleated cells, while T and B lymphocytes, eosinophils, and natural killer (NK) cells were detectable at frequencies of <10% each. The majority of urinary T cells showed phenotypical characteristics of activated effector memory T cells (EM) as indicated by the co-expression of CD38 and CD69 – a phenotype that was not detectable in PB. Kidney inflammation was also reflected by tissue-imprinted macrophages, which phenotypically differed from PB monocytes by an increased expression of HLA-DR and CD11c. The presence of activated urinary T cells and macrophages could be used for differential diagnosis of proliferative LN forms and other renal pathologies. Most interestingly, the amount of EM in the urine sediment could be used as a biomarker to stratify LN patients in terms of response to induction therapy. Deep immunophenotypic profiling of urinary cells in LN allowed us to identify a signature of activated T cells and macrophages, which appear to reflect leukocytic infiltrates in the kidney. This explorative study has not only confirmed but also extended the knowledge about urinary cells as a future non-invasive biomarker platform for diagnosis and precision medicine in inflammatory renal diseases.

## Introduction

Systemic lupus erythematosus (SLE) is a rare potentially life-threatening autoimmune disease with a complex and not fully understood immune pathophysiology (1).

Almost half of SLE patients develop lupus nephritis (LN) (2), which is responsible for a significant increase in mortality (18% over 5 years) and morbidity (end-stage renal disease, cardiovascular events) (3). There are several types of renal diseases in SLE, which are mainly immune complex-mediated glomerulonephritis. These can be differentiated by histopathological examination of renal biopsies. Morphological changes are divided into six different classes according to the International Society of Nephrology/Renal Pathology Society (ISN/RPS) 2003 classification of LN and are critical to the issue of patient care (4, 5). While class I and class II are characterized by pure mesangial involvement and do not need specific therapy, class III and class IV LN present with focal (III) or diffuse (IV) endocapillary proliferation and are often treated with potent immunosuppression. Finally, class V membranous glomerulonephritis and class VI, presenting with advanced sclerotic lesions, may be managed conservatively with antiproteinuric, renoprotective measures (6). Combinations of proliferative (III or IV) and membranous (V) lesions can be observed. In addition, renal diseases as pauci-immune glomerulonephritis may be rarely seen in SLE patients.

When LN is suspected by an increased serum creatinine level, new onset proteinuria, erythrocyturia with the occurrence of urinary acanthocytes or casts, a kidney biopsy is indispensable to confirm the diagnosis and define the nature of renal involvement (2). However, several clinical situations might occur in which kidney biopsy is contraindicated, due to severe hypertension, bleeding diathesis, or solitary kidney, or is not performed, such as in mild proteinuria and silent LN (7). In these cases, the diagnosis is uncertain and initiation of induction therapy might be delayed, potentially resulting in ongoing inflammation and subsequent chronic organ damage, while early diagnosed and treated LN patients have a better outcome (8, 9). Moreover, when response to induction therapy has not been achieved after 6 months and a change in the histopathologic classification of the kidney or chronic renal damage is suspected, additional biopsies are required to aid therapeutic decision-making and predicting outcomes (2). In fact, laboratory parameters on which current remission criteria are based (10), especially serum creatinine and proteinuria, cannot distinguish between active disease and established organ damage, and several studies showed their poor sensitivity for predicting disease outcomes (11, 12).

To overcome the limitations of kidney biopsy and to improve LN prognosis, reliable non-invasive biomarkers for early diagnosis and outcome prediction are required. For this purpose, the urine sediment of LN patients was already investigated by conventional flow cytometry before, and the presence of mononuclear cells in urine of patients with active renal disease was described (13–15). Remarkably, these studies showed that the phenotype of urinary immune cells differs from that of peripheral blood (PB) leukocytes while it resembles that of kidney-infiltrating inflammatory cells, suggesting that urinary leukocytes most likely originate from the leukocytic kidney infiltrate intrarenal resident cells and not from blood leaking into urine (12–15). In particular, urinary T cells were revealed as promising biosensors to identify an active proliferative LN. While their frequencies correlated with the extent of renal inflammation, the disappearance of urinary T cells under induction therapy was associated with better outcome. SLE patients with inactive LN or non-proliferative LN forms showed an “immunologically inactive” urine sediment (13, 14). These results support the initial hypothesis that urinary cells yield reliable biomarkers for clinical use.

Conventional flow cytometry has been employed in a hypothesis-based manner to analyze urine with respect to particular cell types. There, not only the limited number of cytometric readouts but also autofluorescence of urinary cells and problems to compensate for spectral spillover artifacts restricted the power of fluorescence-based analyses. Therefore, we have used for the first time fluorescence-free mass cytometry (cytometry by time-of-flight, CyTOF) to systematically map all major and many minor leukocyte subsets typically detectable in PB at unprecedented detail in human urine. This technology uses metal isotope-conjugated monoclonal antibodies to detect for today up to 50 parameters simultaneously at the single-cell level and circumvents the technical limitations of conventional flow cytometry mentioned before (16). This technology allowed us an in-depth analysis of leukocytes of urine with respect to their particular phenotypes associated to cellular differentiation and activation. The identification of a robust cell signature in urine is of potential interest as a non-invasive biomarker for the diagnosis of LN.

## Materials and Methods

### Study Population

Urine and PB samples were collected from patients fulfilling at least 4 of the 2010 American College of Rheumatology revised criteria for SLE (17) and suspected active renal involvement. Active renal involvement was suspected clinically by worsening kidney function, abnormal proteinuria, and/or erythrocyturia of unknown origin. All SLE patients (*n* = 16) underwent renal biopsy and were diagnosed with active LN. Histological findings were evaluated according to the ISN/RPS 2003 classification of LN (4). One patient (P12) was diagnosed with a focal LN (class III), nine patients were diagnosed with a diffuse LN (class IV), three patients (P01, P10, and P15) were diagnosed with mixed proliferative and membranous LN (class IV + V), one patient (P05) was diagnosed with pure membranous LN (class V), and two patients (P07 and P08) showed a pauci-immune, crescentic glomerulonephritis (Table 1).

**TABLE 1:** Baseline characteristics and medication of all included patients.

Patient ID	Sex Age	ISN/RPS class LN/Other GN	SLEDAI	renal SLEDAI	Active urine sediment	Proteinuria (mg/24h)	Creatinine (mg/dl)	Immunosuppressive therapies
01	F, 34	IV - G(A/C) + V	12	8	no	907	0.72	AZA, HCQ
02	F, 31	IV - S (C)	10	8	yes	500	3.49	AZA, HCQ
03	F, 20	IV - G(A)	14	8	yes	393	1.69	
04	F, 29	IV	*11*	12	no	4327	0.91	
05	F, 46	V	8	4	no	1254	0.74	MMF, HCQ
06	F, 38	IV - G(A)	10	8	no	4812	0.74	HCQ
07	F, 51	pauci-immune*	13	8	yes	1424	1.17	CS
08	F, 33	pauci-immune** + V	12	8	yes	1019	1.69	
09	F, 25	IV	18	12	yes	1584	0.62	
10	F, 30	IV - G(A) + V	16	12	yes	2994	2.45	
11	F, 19	IV - G(A/C)	22	16	yes	1264	0.79	HCQ
12	F, 33	III - S(A)	26	16	yes	637	0.79	CS
13	F, 24	IV - G(A/C)	12	8	no	654	0.80	AZA, HCQ
14	F, 40	IV - G(A)	13	8	no	1097	0.67	AZA, Belimumab
15	F, 56	IV - G(A/C) + V	18	12	yes	3384	2.79	AZA
16	F, 20	IV - S(A)	14	8	yes	393	1.89	
17	M,40	AAV	n.a.	n.a.	yes	568	4.63	
18	F, 29	gITN	n.a.	n.a.	no	128	4.44	
19	F, 64	AAV	n.a.	n.a.	yes	76	0.92	

Proliferative LN cohort in white; control cohort in gray. *Focal necrotizing, crescentic glomerulonephritis by SLE. **Focal crescentic and sclerotic glomerulonephritis by SLE, later by antineutrophil cytoplasmic antibody positivity als LN overlap AAV diagnosed. LN, lupus nephritis; GN, glomerulonephritis; G, global; S, segmental; A, active, C, chronic lesions; AAV, ANCA-associated glomerulonephritis; gITN; granulomatous interstitial tubulonephritis; SLEDAI, Systemic Lupus Erythematosus Disease Activity Index; AZA, azathioprine; HCQ, hydroxycloroquine; MMF, mycophenolate mofetil; CS, corticosteroids.

In a subgroup of 13 patients with proliferative LN (LN class III, IV, and mixed forms), paired urine and blood samples were analyzed to identify proliferative LN-specific urinary biomarkers. For P01 und P16, no blood samples were available, so urine samples were analyzed only. Samples from SLE patients with pauci-immune LN forms (P07 and P08), with biopsy-proven anti-neutrophil cytoplasmic antibodies (ANCA)-associated glomerulonephritis (P17 and P19) and with acute granulomatous interstitial tubulonephritis (gITN; P18) were used as non-proliferative control group (Table 1). P05 (class V) was not used for further analysis, because relatively low numbers of urine cells were detectable and most of them were granulocytes.

For each SLE patient, SLE Disease Activity Index (SLEDAI) (18) was assessed; SLEDAI elements accounting for kidney disease (proteinuria, leukocyturia, erythrocyturia, and casts) are presented as renal SLEDAI, whereby each positive element is weighted with four points. Routine laboratory values as well as urine sediment findings were retrieved from the medical records.

All patients with proliferative LN received induction therapy with combinations of high-dose corticosteroids (>1 mg/kg body weight) and either cyclophosphamide (CYC) or mycophenolate mofetil (MMF) for 6 months (in case of patient 03 only 3 months) (Table 2). Patients were clinically monitored for 6 months. At this time, the response to induction therapy was assessed by adopting a simplified version of the ACR renal response criteria and verifying clinician’s judgment (Table 2). We defined patients as responders when creatinine remained stable (if estimated glomerular filtration rate (eGFR) was >60 ml/min at baseline) or improved of at least 50% (if eGFR was <60 ml/min at baseline), and/or proteinuria showed an improvement of at least 50% without worsening of other findings (creatinine, proteinuria, and urine sediment/hematuria) over time. Patients who did not fulfill these criteria were considered as non-responders. According to this assessment, nine patients were responders and four were non-responders (Table 2).

**TABLE 2:** Clinical and laboratory response to induction therapy.

Patient ID	Induction Therapy*	Creatinine [mg/dl); eGFR		Proteinuria^#^		Clinical valuation	Response to Treatment
		Before	After	Before	After		
01	CS+ CYC	0.72; >90	0.55; >90	907	204	good response to CYC, switch to maintenance therapy	R
02	CS+ CYC	3.55; 16	3.13; 19	505	220	proteinuria improved, stabile GFR by chronic renal failure	R
03	CS+ CYC (3 months)	1.69; 43	0.64; >90	393	42	good response so far (after 3 months)	R
04	CS+ CYC	0.91; 73	0.76; >90	4327	231	good response, switch to maintenance therapy	R
06	CS+MMF	0.74; >90	n.d.	4812	>3000	active disease, induction therapy has to be continued	NR
09	CS+ CYC	0.62; >90	0.68; >90	1584	228	no more disease activity, switch to maintenance therapy	R
10	CS+MMF	2.45; 26	0.85; >90	3151^#^	610^#^	good response to induction therapy	R
11	CS+ CYC	0.79; 89	0.55; >90	1264	1074	active disease, switch to MMF	NR
12	CS+MMF	0.56; >90	0.71; >90	668	189^#^	good response to MMF, switch to maintenance therapy	R
13	CS+ CYC	0.80; >90	0.93; 86	654	1527	worsening of proteinuria, switch to MMF	NR
14	CS+MMF	0.67; >90	0.86; 87	535^#^	272^#^	good response to induction	R
15	CS+ CYC	2.79; 18	1.40; 48	3384	1651	incomplete response to CYC, switch to MMF	NR
16	CS+ CYC	1.89; 38	0.62; >90	617^#^	108^#^	good response, switch to maintenance therapy	R

*Induction therapy was given for 6 months (except for Patient P 03) Proteinuria is expressed as mg/24h or as uPCR. uPCR values are labeled with #. eGFR, estimated Glomerular Filtration Rate (ml/min); uPCR, urinary Protein Creatinin Ratio (mg Protein/mg Creatinine); n.d., not determined; R, response; NR, non-response; CS, corticosteroids; CYC, cyclophosphamide; MMF, mycophenolate mofetil.

All patients were recruited in a time period of 4 years (between 2014 and 2017) from the ward of the Department of Rheumatology and Clinical Immunology, Charité University Hospital, Berlin, Germany. Informed consent was obtained from all patients. The study was approved by the ethics committee of the Charité University Hospital (EA1/356/14) and was conducted in accordance with the Declaration of Helsinki.

### Sample Preparation

#### Buffers, Chemicals, and Materials

The following buffers and chemicals were used: erythrocyte lysis buffer (Qiagen, Hilden, Germany), 1 × PBS, made from 10 × PBS (Rockland, Gilbertsville, PA, United States; pH 7.2) using Millipore water, 0.1% cell permeabilization buffer, made from 10 × saponin-based permeabilization buffer (eBioscience), 4% formaldehyde solution in PBS made from 16% paraformaldehyde (EMS, Hatfield, PA, United States), PBS supplemented with 0.5% BSA (PAN Biotech, Aidenbach, Germany), and 0.02% sodium azide (Sigma-Aldrich, St. Louis, MO, United States) (PBS/BSA). Buffers were sterile-filtered through 0.22-μm membranes and stored in Stericup disposable bottles (Merck, Darmstadt, Germany). Blood, urine, and cells were processed in 50-ml, 15-ml, and 5-ml round-bottom polystyrene/polypropylene tubes (Corning, Corning, NY, United States and Sarstedt, Nümbrecht, Germany).

MAXPAR antibody labeling kits, EQ Four element calibration beads, washing and tuning solution, and DNA intercalators were purchased from Fluidigm Corporation (South San Francisco, CA, United States). Pre-conjugated and unlabeled antibodies are summarized in the Supplementary Table S1.

*Cis*-Platinum (II)-diamine dichloride (cisplatin) was purchased from Enzo Life Sciences GmbH (Lörrach, Germany). A 25 mM stock solution was prepared in DMSO (Sigma-Aldrich) and aliquots were stored at -20°C.

#### Sample Collection and Preservation of Urinary Cells

EDTA anti-coagulated blood and fresh voided urine samples (50 to 150 ml) were collected from each patient. Patients with active urinary infections or menstruation at the time of urine collection were excluded. Fresh urine was diluted with 30% v/v PBS supplemented with 1% BSA and promptly centrifuged (300 × *g*, 10 min, 4°C) within 60 min after collection in order to minimize artifacts resulting from delayed sample processing. Urinary cells were washed twice with PBS/BSA. Blood was subjected to erythrocyte lysis within 1 h after collection. One volume of whole blood was mixed with 4 volumes of EL buffer, incubated for 10 min on ice, and centrifuged (300 × *g*, 10 min, 4°C), and cells were washed once with EL buffer and twice with PBS/BSA.

The number of leukocytes and their viability were checked by conventional flow cytometry staining successively with CD45 (Miltenyi Biotec; Vio770 conjugate diluted 1:50) and 4,6-diamidino-2-phenylindole dihydrochloride (DAPI) (Sigma-Aldrich; final concentration 1 μg/ml) just before measuring on a MACSQuant flow cytometer (Miltenyi Biotec, Bergisch-Gladbach, Germany). Urine samples with at least 1 × 10^5^ viable CD45-positive cells were used for mass cytometric analysis. While the viability of blood cells was always >95%, a large variability was seen for urine cells, which ranged from 5% to 55% (mean 33%; SD ± 29%).

#### Sample Processing for Mass Cytometric Analysis

Up to 3 × 10^6^ cells were incubated with cisplatin as described before to stain dead cells. A cocktail including 26 anti-human monoclonal antibodies (Supplementary Table S1) was used to stain blood and urinary cells for 30 min at room temperature as described before (19). All in-house-conjugated antibodies were titrated before using blood cells. Commercial antibodies were used as recommended by the manufacturer.

After washing, cells were fixed overnight using 4% PFA. On the next day, samples were washed and nucleated cells were stained with iridium intercalator in permeabilization buffer for 30 min at RT. After washing twice with PBS/BSA and subsequently twice with Millipore water, cells were adjusted to 0.5 × 10^6^ cells/ml using Millipore water supplemented with 1/10 v/v EQ Four element beads.

### Mass Cytometric Measurement

Cell suspensions were acquired on a CyTOF v1 instrument controlled by CyTOF software v5.1.6.4.8 and v6.0.626) (Fluidigm). The instrument was set up and tuned daily according to the manufacturer’s recommendations. Cells were injected into a 450-μl loop at a flow rate of 45 μl/min. Data were acquired in dual calibration mode, with noise reduction turned on and lower and upper cell length parameter thresholds set to 10 and 75. Absolute cell numbers of each particular sample are summarized in Supplementary Figure S1. For blood samples, about 2.5 × 10^6^ total events were acquired.

### Data Analysis and Statistics

Data were normalized based on signals of the internal standard beads. Zero values were randomized to values between -1 and 0. Data were then analyzed by manual gating using FlowJo 10.6 software (Treestar, Ashland, OR, United States). Blood and urinary leukocytes were identified by the expression of CD45. The gating strategy applied to urine and blood samples is shown in Supplementary Figure S2. viSNE analyses of paired urine and blood samples were performed by Cytobank premium (Santa Clara, CA, United States)^1^ using the following parameters: perplexity = 100, theta = 0.5, iterations = 1000.

Statistical analyses were conducted using GraphPad Prism 8.0 (GraphPad Software, San Diego, United States). Non-parametric Wilcoxon test was used for paired analyses of blood and urine. Correlation analyses with disease activity were assessed using Spearman’s rank correlation. Non-parametric Mann–Whitney *U* test was adopted to analyze differences between responder and non-responder groups. Two-tailed *p*-values of less than 0.05 were considered statistically significant. The hierarchical cluster analysis was generated based on *z*-score standardized frequencies of cell subpopulations obtained from urine samples of patients with active LN and other acute renal diseases. Cell frequencies were obtained by manual gating of mass cytometric data. Hierarchical clustering was performed based on Spearman distance and the Ward linkage criterion.

## Results

### Identification of Viable, Nucleated Cells in PB and Urine

At first, the presence of live, single nucleated cells was evaluated according to the manual gating strategy exemplarily shown for PB and urine cells in Supplementary Figure S2. While stable amounts of live single CD45^+^ cells were recovered from PB samples, corresponding urine samples of LN patients showed large variations with respect to cell density in the urine and in cell viability ranging from 5% to 55%. In Supplementary Figure S1 absolute numbers of nucleated cells acquired by mass cytometry are summarized.

### Which Immune Cells Can Be Found in Urine of LN Patients?

**FIGURE 1:**
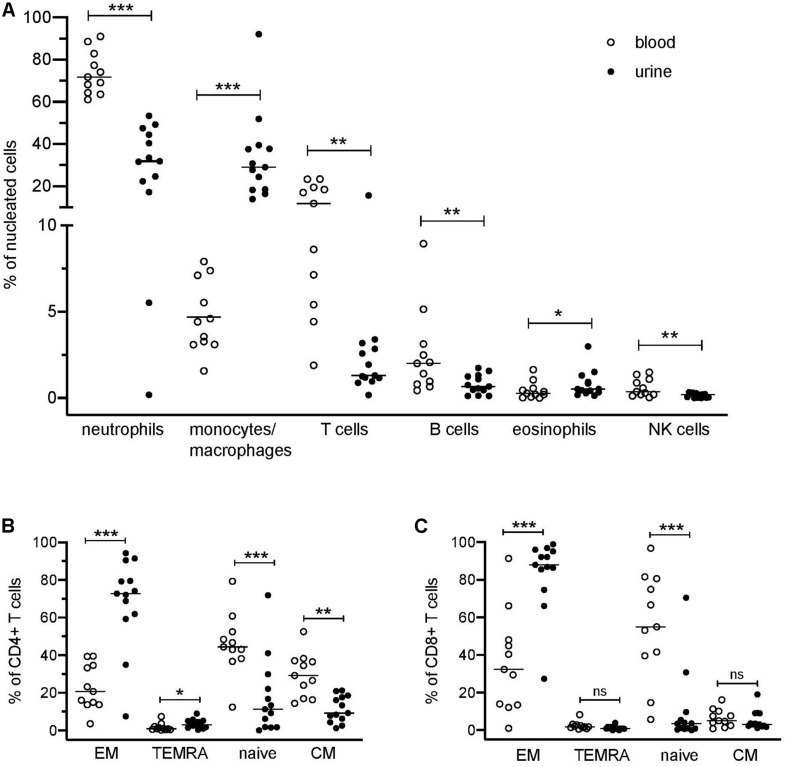
Distribution of blood and urinary immune cells. **(A)** Frequencies of urinary leukocytes (% of nucleated cells) in comparison to blood. **(B)** Frequencies of CD4^+^ naïve and effector cell subsets in urine and blood (% of CD4^+^ T cells). **(C)** Frequencies of CD8^+^ naïve and effector cell subsets in urine and blood (% of CD8^+^ cells). EM, effector memory T cells; TEMRA, terminally differentiated effector memory T cells; CM, central memory T cells. Wilcoxon test for paired samples, *p*-values: *p < 0.05, **p < 0.01, ***p < 0.001, n.s.: not significant.

The application of a comprehensive mass cytometry antibody panel allowed an in-depth analysis of the urinary leukocyte composition in comparison to autologous blood samples. Neutrophils, monocytes/macrophages, and T lymphocytes were identified as predominating leukocyte populations in the urine, adding to 32–84% of all nucleated cells (Figure 1A). In addition, minor frequencies (<4% of nucleated cells) of B lymphocytes, eosinophils, and natural killer (NK) cells were detectable (Figure 1A).

In contrast to the paired PB samples, no reasonable numbers of dendritic cells, basophils, or plasma cells/plasmablasts were ascertainable in urine samples. If compared to PB samples, a large variability was seen in the cellular composition of the urine cell sediment regarding neutrophils (17–53% of nucleated cells), monocytes/macrophages (12–78% of nucleated cells), and T cells (0.2–16% of nucleated cells) (Figure 1A). In the Supplementary Table S2 all phenotypes identified by the manual gating are summarized.

The results obtained by manual gating could be confirmed by t-SNE analysis, which revealed similar cell clusters (Figure 3B). The most obvious difference between PB and urine was seen within the myeloid compartments. Here clusters of granulocytes and monocytes/macrophages showed clear changes in their spatial classification. The changed phenotype of granulocytes seen in urine samples most probably reflects their sensitive behavior in response to the chemical nature of urine.

### Can Urine-Specific Immune Cell Phenotypes Be Identified?

Beyond the abundance of major leukocyte subsets, we further analyzed the immunophenotypic characteristics of urinary T cells and monocytes in more detail. Urinary T cells showed a significant lower CD4/CD8 ratio as compared to the paired PB samples (0.8 ± 0.8 vs. 1.6 ± 0.7, *p* = 0.02) and only a poor correlation between blood and urine was observed (*r* = 0.2760, *p* = n.s.).

T cells lacking CD4 and CD8 (DN) were significantly increased in urine as compared to blood (28% ± 13% vs. 8% ± 5% of CD3^+^ cells; *p* = 0.002).

Naïve (CD45RA^+^CCR7^+^), central memory (CM, CD45RA^–^CCR7^+^), effector memory (EM, CD45RA^–^CCR7^–^), and terminally differentiated effector memory (TEMRA, CD45RA^+^CCR7^–^) T cell subsets showed a significantly different distribution in urine and blood (Figures 1B,C). In urine, both CD4^+^ and CD8^+^ effector memory T cells represented by far the largest T cell subset (CD4^+^ EM, 68% ± 24%; CD8^+^ EM, 84% ± 19% of CD4^+^ and CD8^+^ cells, respectively), while in PB, naïve T cells were dominating (CD4^+^ naïve: 46% ± 16%; CD8^+^ naïve: 55% ± 28% in relation to all CD4^+^ and CD8^+^ cells, respectively).

**FIGURE 2:**
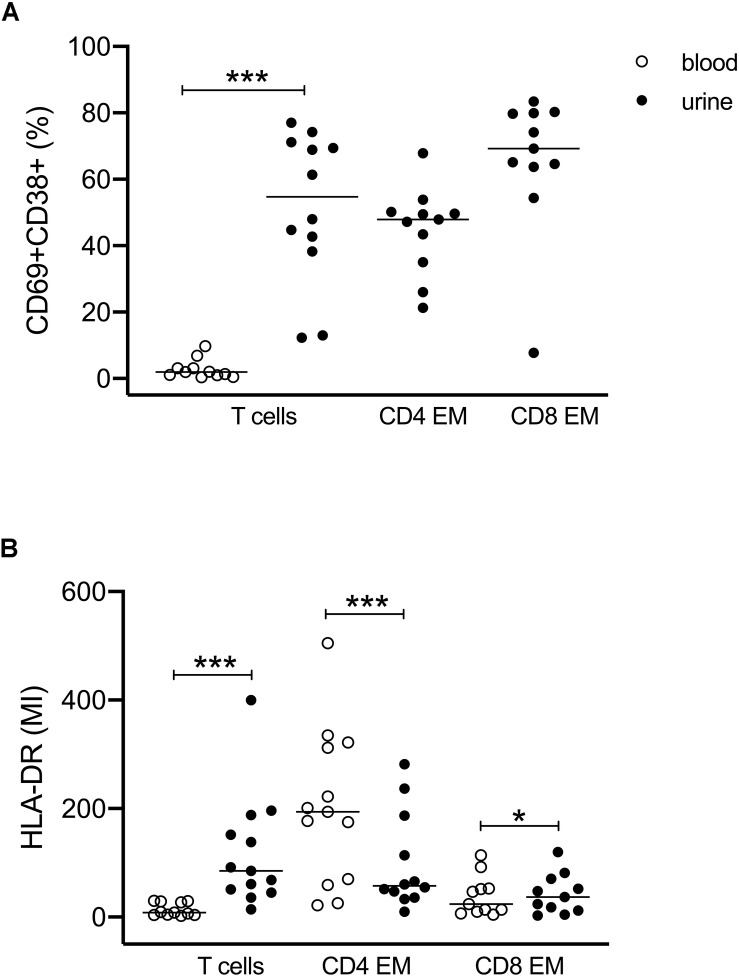
Activated T cells in urine and blood. **(A)** Frequencies of CD69^+^CD38^+^ double-positive T cell subsets related to all CD3^+^ and to CD4^+^ EM and CD8^+^ EM cell subsets. Since almost no activated T cells were found in blood, CD4- and CD8-related analyses were shown for urinary cells only (closed symbols). **(B)** Mean intensity of HLA-DR expression on all CD3^+^, CD4^+^ EM, and CD8^+^ EM cells in urine (closed symbols) and blood (open symbols). Wilcoxon test for paired samples, *p*-values: *p < 0.05, ***p < 0.001.

We further investigated the expression of activation markers on particular T cell subsets, i.e., HLA-DR, CD69, and CD38. The majority of urinary T cells showed a CD69^+^CD38^+^ double-positive phenotype (52% ± 23% of CD3^+^ cells), while this phenotype was almost absent in blood (3% ± 3%) (Figure 2A). The percentage of CD69^+^CD38^+^ T cells was highest among CD8^+^ EM cells (66% ± 21%) followed by CD4^+^ EM cells (45% ± 13%) (Figure 2A).

The activated phenotype of urinary T cells was also reflected by a higher expression of HLA-DR (Figure 2B). Here, CD4^+^ EM cells showed twofold higher mean intensity values than CD8^+^ EM cells.

**FIGURE 3:**
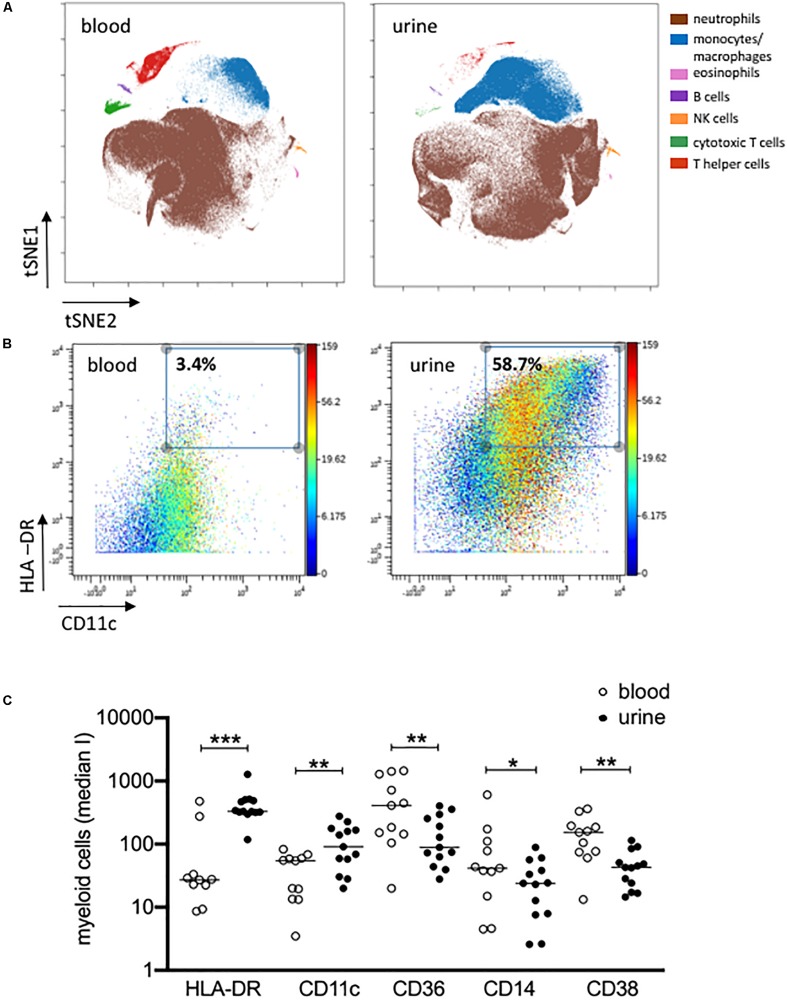
Activated monocytes/macrophages in urine and blood. **(A)** viSNE analysis of all pre-gated viable nucleated cells from urine and blood (plot 5, Supplementary Figure S1) with cell populations defined based on basic phenotypic markers exemplarily shown for patient 04 (equal sampling per comparison, event sampling = 200,000 cells per sample; 1,000 iterations, final KL divergence: 5.11). **(B)** HLA-DR and CD11c expression on pre-gated monocyte/macrophages cell population shown in a two-dimensional plot; color corresponds to CD14 expression exemplarily shown for patient 04 in urine and blood. **(C)** Myeloid cells gated before for HLA-DR and CD11c were further analyzed for the expression of HLA-DR, CD11c, CD36, CD14 and CD38 as median intensities (median I). Wilcoxon test for paired samples, *p*-values: *p < 0.05; **p < 0.01; ***p < 0.001.

The results obtained by manual gating could be confirmed by t-SNE analysis, which revealed similar cell clusters (Figure 3A). The most obvious difference between PB and urine was seen within the myeloid compartments. Here, clusters of granulocytes and monocytes/macrophages showed clear changes in their spatial classification. The changed phenotype of granulocytes seen in urine samples most probably reflects their sensitive behavior in response to the chemical nature of urine. t-SNE analyses allowed an in-depth phenotypic characterization of the monocyte/macrophage clusters exemplarily shown in Figure 3B. While monocytes were detectable by the co-expression of CD14, CD36, CD16, and HLA-DR in PB, obviously tissue-imprinted macrophages were characterized by high expression values for HLA-DR and CD11c in urine (Figures 3A,B). CD36, CD14, and CD38 were significantly lower expressed on urine macrophages as compared to PB monocytes (Figure 3C and Supplementary Figure S3).

### Can a Urinary Leukocyte Signature Be Used to Identify Proliferative LN Patients?

**FIGURE 4:**
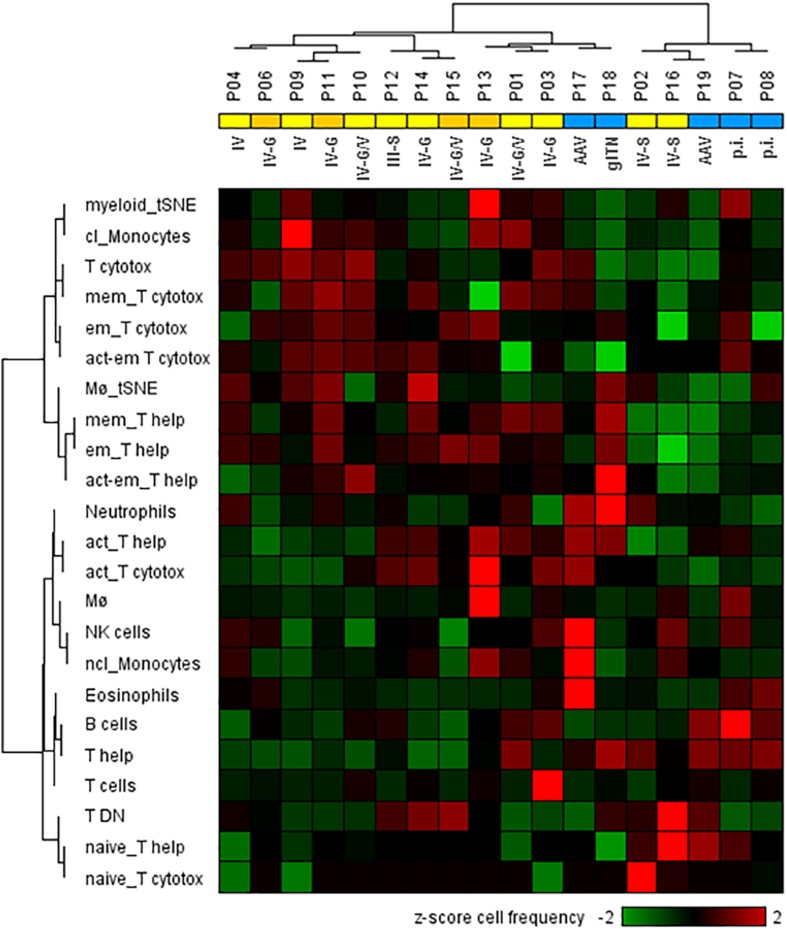
Two-dimensional hierarchical cluster analysis using 23 urinary cellular phenotypes for the classification of active proliferative LN (*n* = 13) and other renal diseases (*n* = 5). The heat map was generated based on *z*-score standardized frequencies of cell subpopulations obtained from urine samples of patients with active LN and other acute renal diseases that were obtained by manual gating of mass cytometric data. Hierarchical clustering was performed based on Spearman distance and Ward linkage criterion. Values above 2 and below -2 standard deviations are highlighted by light red and green, respectively. Proliferative LN patients who did not respond to induction therapy were marked in dark yellow. Analysis revealed two main clusters of patients consistent with histological results for proliferative LN patients (yellow) and kidney disorders with different diagnoses (blue). However, two proliferative LN patients clustered within the control group and two controls clustered in the proliferative LN group. Abbreviations according to Table 1 and Supplementary Table S2 respectively, p.i.: pauci-immune.

Next, we analyzed whether the cellular phenotypes identified in the urine of LN patients can be used to distinguish proliferative from non-proliferative LN forms and other acute inflammatory renal diseases. For this analysis, cell populations were considered when at least 25 cell counts were detectable; i.e., CM, TEMRA cells, Treg’s, and DC’s were excluded. This was the reason why P05 (membranous LN) was omitted from analysis because, here, urinary neutrophils were almost exclusively detected. As shown in the hierarchical cluster analysis of Figure 4, the frequencies of 23 urinary leukocyte subsets as summarized in the Supplementary Table S2 allowed a separation of patients into two main clusters. This classification is largely in agreement with histopathological evaluation of kidney biopsies. In fact, 11 out of 13 proliferative LN samples clustered together and clearly separated from the other ones (Figure 4). Interestingly, P02 and P16, which clustered separately together with non-lupus samples, were the only class IV patients showing segmental lesions in the glomeruli instead of global lesions observed in all the other class IV patients. Two of five controls (P17 and P18) clustered together with proliferative LN samples.

### Do Urinary T Cell Subsets Correlate With Clinical Parameters?

Next, we investigated which urinary cell subsets correlate with clinical parameters. The only significant correlation observed was between CD4^+^ T cells and proteinuria. Here, a negative correlation could be ascertained, which reflects the low CD4/CD8 ratio detected in PB and urine of LN patients (Supplementary Figure S4). Other clinical parameters such as renal SLEDAI, active urinary sediment, and creatinine did not show significant correlations with any of the cell subsets identified.

### Can Urinary Cell Signatures Be Used as Therapy Response Predictors?

**FIGURE 5:**
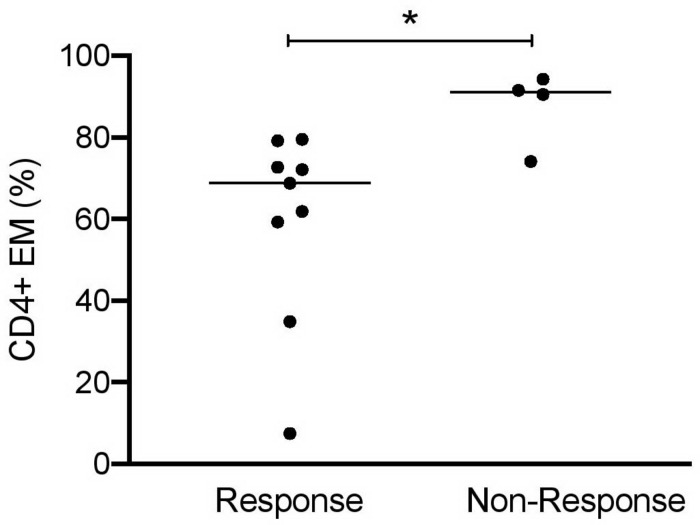
CD4^+^ EM cells as predictors of responsiveness at baseline of induction therapy in active proliferative LN patients (*n* = 13). Frequencies of effector memory CD4^+^ cells at baseline in patient responder and non-responder groups. Responsiveness was calculated 6 months after starting induction therapy as described in Table 2. Mann–Whitney test for unpaired samples, *p*-value: *p < 0.05.

Finally, it was interesting to know whether urinary cell subsets can be used to predict response to induction therapy. LN patients were classified 6 months after starting induction therapy as responders or non-responders (Table 2). In 9 out of 13 LN patients, clinical improvement was observed in response to standard immunosuppressive therapies. High frequencies of CD4^+^ EM cells (>90% of total CD4^+^) were identified at baseline as the most promising predictor to indicate insufficient response to therapy (Figure 5). All other urinary cell subsets did not allow prediction of therapy response or non-response.

With regard to PB, we could not identify any leukocyte subset predictive for therapy response.

## Discussion

The availability of non-invasive biomarkers in LN, which could be diagnostically used to clarify renal injury and which would help in individualized therapeutic decision-making, is hitherto unmet clinical needs. Therefore, this study used state-of-the-art mass cytometry to enable an in-depth profiling of urinary leukocytes in comparison to PB with respect to identifying new cell-associated biomarkers that can be used along with humoral markers of serum and urine for an innovative, non-invasive management of LN.

Although the cellular composition of the urine sediment has already been analyzed in several studies (13–15, 20–23), these commonly fluorescence-based flow cytometric analyses were restricted by methodological limitations, such as limited number of parameters necessary for deep and comprehensive profiling of leukocyte subsets, issues emerging from autofluorescence of cells present in the urine, and how these can be appropriately compensated for when spilled over in the fluorescence channels of interest. As mass cytometry eliminates these technical limitations, we have used it for a deep comparative profiling of blood and urinary leukocytes. So, we were able to identify significant differences in the distribution of leukocyte subsets and phenotypic features, which are exclusively expressed by urinary cells, supporting the assumption that urinary leukocytes may reflect the inflammatory infiltrate in LN. This assumption is supported by data of a recent study analyzing urine and kidney-related cell clusters in LN by single-cell transcriptomics (24). Here, different macrophage subsets, NK cells, and lymphocyte subsets were identified in urine, which were also assignable to tissue-located leukocytes in the inflamed kidney. Contrary to the study of Arazi et al. (24), our mass cytometry approach allowed the detection of neutrophils, which, along with monocytes/macrophages, are the dominant leukocyte subsets detectable in the urine sediment and, consequently, have a strong influence on the relative quantitative composition of urinary cells.

With the present data, we could not only confirm the occurrence of main inflammatory cell types in the urine from affected kidneys but also detect rare cell types such as B cells, NK cells, or eosinophils at the single-cell level. With the exception of Brito’s investigation of urinary eosinophils (20), most studies conducted so far focused on mononuclear cell subsets, although they account for only 20% of total cells detectable in urine of LN patients (21). Several recent publications indicate that granulocytes (neutrophils, basophils, and eosinophils) play an important role in SLE pathogenesis by promoting innate and adaptive aberrant autoimmune responses and enhancing tissue damage (25). Therefore, our antibody panel was conceived to investigate both granulocytic and mononuclear cell populations. According to our data, neutrophilic granulocytes and mononuclear cells account for roughly 50% of nucleated cells in the urine. Comparing neutrophils from urine and blood, phenotypic differences were detectable according to the t-SNE analyses. It was not possible to assign clearly defined phenotypes since the granulocytic compartment showed high inter-individual differences, which may be induced by the chemical nature of the urine. Obviously, granulocytes are particularly very sensitive to this kind of cell stress and apoptotic programs will be initiated.

The second largest urinary cell population consisted of monocytes/macrophages. When compared to blood monocytes, urinary cells showed a more differentiated macrophage-like phenotype as indicated by an increased expression of HLA-DR and CD11c (26).

T cells are most frequently investigated in LN urine (21–23). The predominant appearance of urinary T cells and monocytes besides granulocytes confirms histopathologic findings of the inflamed tissue in LN (22, 27–29), again supporting the view that leukocytes attracted into the inflamed kidney may escape from damaged glomerular capillaries and tubules into the urine. Our data allowed an in-depth phenotyping of urinary T cells, which have been already described as reliable biomarkers in proliferative LN (14). In line with the findings of Dolff et al. (15), we could confirm that urinary T cells have predominantly an effector memory phenotype, but additionally could show that they co-expressed the activation markers CD38 and CD69. Comparable results were obtained by single-cell transcriptome analyses of LN kidneys, in which CD69-positive central memory and effector memory T helper cells were identified as one of the main lymphocyte clusters (24). Most strikingly, the amount of effector memory T cells was predictive for the response to immunosuppressive induction therapy. Furthermore, most of the CD4^+^ EM cells could be identified as CCR5-expressing Th1 cells in a subgroup of patients analyzed by a second antibody panel (Supplementary Figure S5), which are known to be primarily responsible for disease progression in LN (30–32).

Besides CD4^+^ and CD8^+^ T cell subsets, double-negative (DN) CD3^+^ T cells were identified in both blood and urine. These cells were discussed to be producers of IL-17 and were found to be increased in glomerulonephritis (33).

Although promising, the expression of this T cell-related activation signature (CD69, CD38, and HLA-DR) did not show any prognostic relevance in our cohort. Instead, it was a striking finding that those patients having high percentages of urinary EM T cells at baseline (especially CD4^+^ EM) were not appropriately responding to therapy.

According to current data, urinary immune cells are not only detectable in active proliferative LN but also present in other inflammatory renal diseases. However, the distribution of leukocyte populations and their respective phenotypes varies among patient groups (22, 23, 34), probably disclosing the differences in the respective renal infiltrates. To show that urinary leukocyte signatures can possibly be used for differential diagnosis of different renal pathologies, we performed a supervised hierarchical cluster analysis including proliferative LN samples as well as non-proliferative LN forms and other acute inflammatory diseases, and could demonstrate that 11 out of 13 proliferative LN samples clustered together and clearly separated from the others. Interestingly, one of both misclassified proliferative LN samples (P16) showed a cellular composition that was very similar to that of LN blood samples and may be caused by a superimposed bleeding derived from an infection of the genitourinary tract. Two of the controls clustered together with proliferative LN samples and therefore the disease specificity of urinary cell signature has to be validated in a larger cohort.

In summary, we could show that the multiplexing power of mass cytometry did allow a more detailed description of urinary T cell and monocyte signatures, disclosing promising biomarker tools for clinical purposes and also revealing some new knowledge about those phenotypes, which drive the inflammation in proliferative LN. Due to the limited number of patients included in this study, we are aware of its explorative nature. Therefore, the conclusions drawn from our results have to be proven by independent and larger cohorts to reliably evaluate the diagnostic and prognostic value of urinary cell signatures for precision medicine in LN patients.

Somehow unclear is the observation that a wide array of dead cells can be found in the urine sediment. On the one hand, the viability is certainly influenced by pH and hyper- or hypo-osmotic nature of the urine, but on the other hand, urinary dead and apoptotic cells may reflect the defective clearance of apoptotic cells in the inflamed kidney, which is unequivocally implicated in the etiopathogenesis of SLE (35, 36). Besides leukocytes, epithelial cells from damaged parenchymal structures, such as glomerula, tubules, and collecting ducts, might also be found in the urine sediment. Accordingly, a more detailed analysis of dead cells with respect to apoptosis-related molecules and epithelial markers might provide additional information about the pathophysiological state of the injured kidney. Nevertheless, we propagate to dilute urine with PBS/BSA buffer immediately after voiding as a simple pre-diagnostic processing procedure to achieve a more standardized collection of urine samples.

In conclusion, urinary cells, being accessible in a non-burdensome manner, would perfectly allow a real-time longitudinal monitoring of cellular changes in the inflamed kidney. It seems to be undisputed that singular markers alone are not likely to have sufficient sensitivity and specificity as required in patients’ clinical management. Hence, urinary multi-parametrical panels including humoral factors, micro RNAs, and cellular markers should be tested in appropriately designed clinical studies. The deeper the knowledge about robustly assessed urinary signatures is, the more likely is the successful establishment of a non-invasive tool for differential diagnosis and prognostic stratification of LN patients.

## Data Availability Statement

The datasets generated for this study are available on request to the corresponding author.

## Ethics Statement

The studies involving human participants were reviewed and approved by the Ethics Committee of the Charité University Hospital (EA1/356/14). The patients/participants provided their written informed consent to participate in this study.

## Author Contributions

MB, SB, and AG wrote the manuscript. MB, SB, PE, HM, and AG have designed the study and the experimental strategy. MB, SB, and PD were responsible for data analysis. MB, TR, and PE were responsible for patient selection and their clinical characterization. SB and AP performed the sample preparations and measurements.

## Conflict of Interest

The authors declare that the research was conducted in the absence of any commercial or financial relationships that could be construed as a potential conflict of interest.

## Footnotes

1 www.cytobank.org
