# Supplementary material for: Deep Phenotyping of Urinary Leukocytes by Mass Cytometry Reveals a Leukocyte Signature for Early and Non-Invasive Prediction of Response to Treatment in Active Lupus Nephritis
Source: Front Immunol. 2020 Mar 24;11:256. doi: 10.3389/fimmu.2020.00256 (PMC7105605; doi:10.3389/fimmu.2020.00256)
Supplement: Supplementary file 1 [file Data_Sheet_1.pdf]

Figure S1: Gating strategy for urinary cells

Exemplary mass cytometric data analysis of urinary (A) and peripheral blood cells (B) (patient 04) by manual gating. Plots 1-3 were successively applied to discriminate single, live cells from cell debris, cell aggregates and dead cells according to Tanner et al., 2013 and Baumgart et al., 2017. Four-element beads added as internal standard to samples prior to data acquisition for later normalization (Finck et al., 2013) were excluded according to their unique ^{140}Ce signal (plot 4). In plot 5 we have gated on all nucleated cells including CD45-positive and negative cells since a clear separation of positive and negative cell subsets was not possible. This is caused by granulocytes, which show a weaker expression of CD45 than other leukocyte subsets and especially in urine samples by other non-leukocytic cells, which do not express CD45, such as epithelial cells.

As next CD3^{+}CD16^{-} T cells (T) were gated in plot 6. They were further divided into CD4^{+}, CD8^{+} and CD4^{-}CD8^{-} double-negative (DN) T cells (plot 11). CD4^{+}CD25^{+}CD127^{low} regulatory T cells (Treg's) were gated proceeding from CD4^{+} T cells (plot 12). For both CD4^{+} and CD8^{+} cells, frequencies of HLA-DR expressing cells (plots 13 and 14, respectively) and frequencies of CD45RA/CD45RO expressing cells were extracted (plots 15 and 16). Furthermore, CD45RA was plotted against CD197 to allow discrimination of CD45RA^{+}CD197^{+} naive, CD45RA^{-}CD197^{+} central memory (CM), CD45RA^{-}CD197^{-} effector memory (EM) and CD45RA^{+}CD197^{-} terminal differentiated effector T cells (TEMRA) (plots 17 and 18, respectively). Activated EM cell subsets were identified by the co-expression of CD38^{+}CD69^{+} (plots 19 and 20, respectively). CD19^{+} and/or CD20^{+} cells were identified as B cells (plot 7). CD3^{-}/CD19^{-}/CD20^{-} -negative cells were used from plot 7 to gate on CD56^{+}/CD16^{+} classical NK cells, which are negative for HLA-DR and CD14 (plot 9).

As next myeloid cells were identified using CD3^{-}/CD19^{-}/CD20^{-}/CD56^{-} cells (plot 8), which were gated for CD15 and CD36 (plot 21). Eosinophilic granulocytes were identified as CD19^{-}CD20^{-}CD36^{-}CD16^{-}CD15^{+}Siglec8^{+} cells after excluding neutrophilic granulocytes (plot 22).

Neutrophilic granulocytes were then depleted by contaminations with HLA-DR-positive cells and were finally read out in plot 24.

Monocytes and dendritic cells were identified according to $CD3^{-}CD19^{-}CD20^{-}CD15^{+/-}$ $CD36^{+}$HLA-$DR^{+/-}$ cells (plot 26), which were finally gated for $CD14^{++}CD16^{-}$ classical and $CD14^{dim}CD16^{+}$ non-classical monocytes (plot 27). Dendritic cell subsets were pre-gated on $CD14^{-}CD16^{-}$ double-negative cells of plot 27. pDCs were finally identified by their expression of $CD123^{+}CD303^{+}$ (plot 29) and mDCs as being $CD123^{-}CD303^{-}$ (plot 29), which were then gated for CD1c and CD11c (plot 30). In the plots 10, 25 and 28 the expression of CD45 is shown for T cells, neutrophils and classical monocytes.

Figure S1A

Manual gating scheme of blood cells (patient 04).

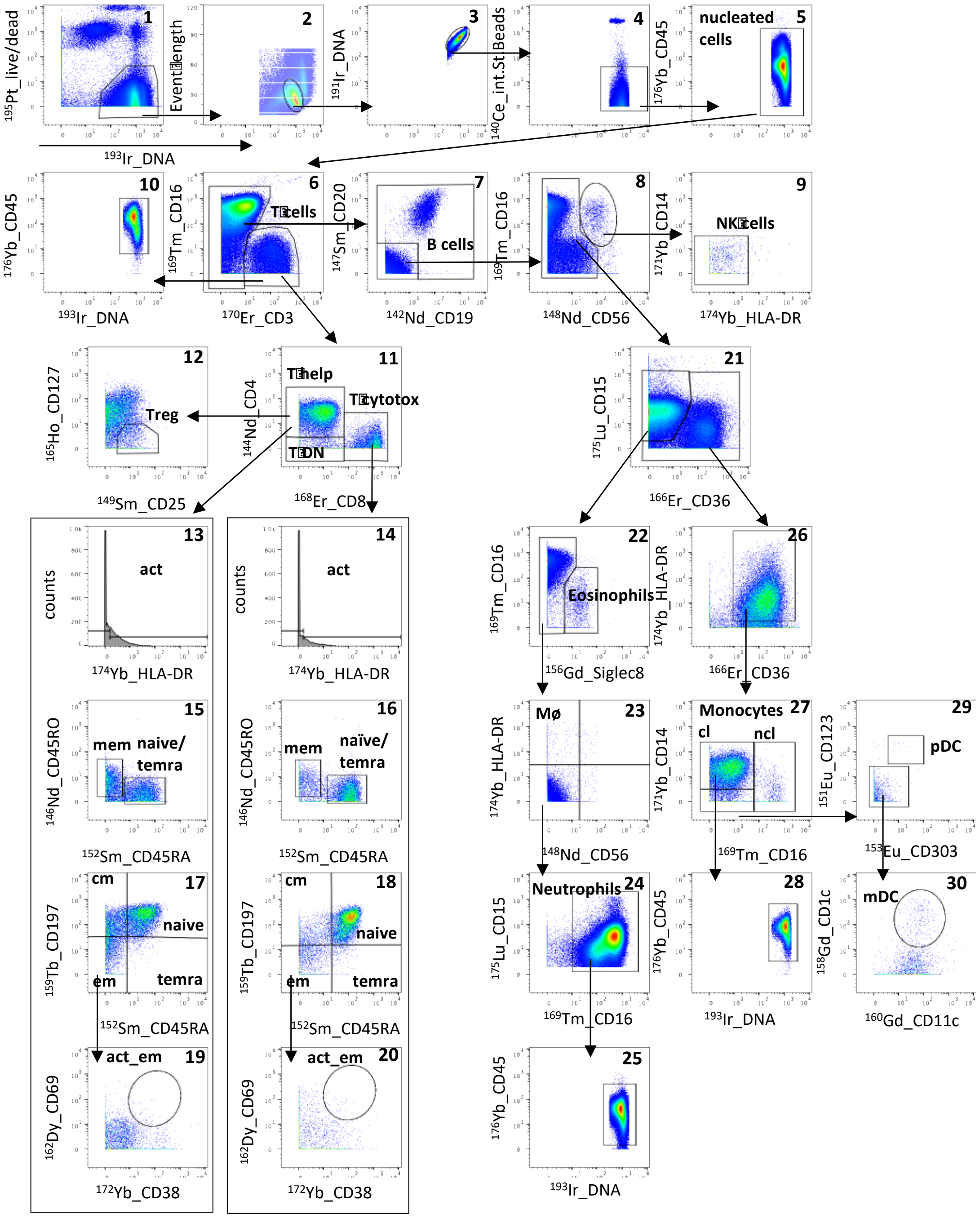

Figure S1B

Manual gating scheme of urine cells (patient 04).

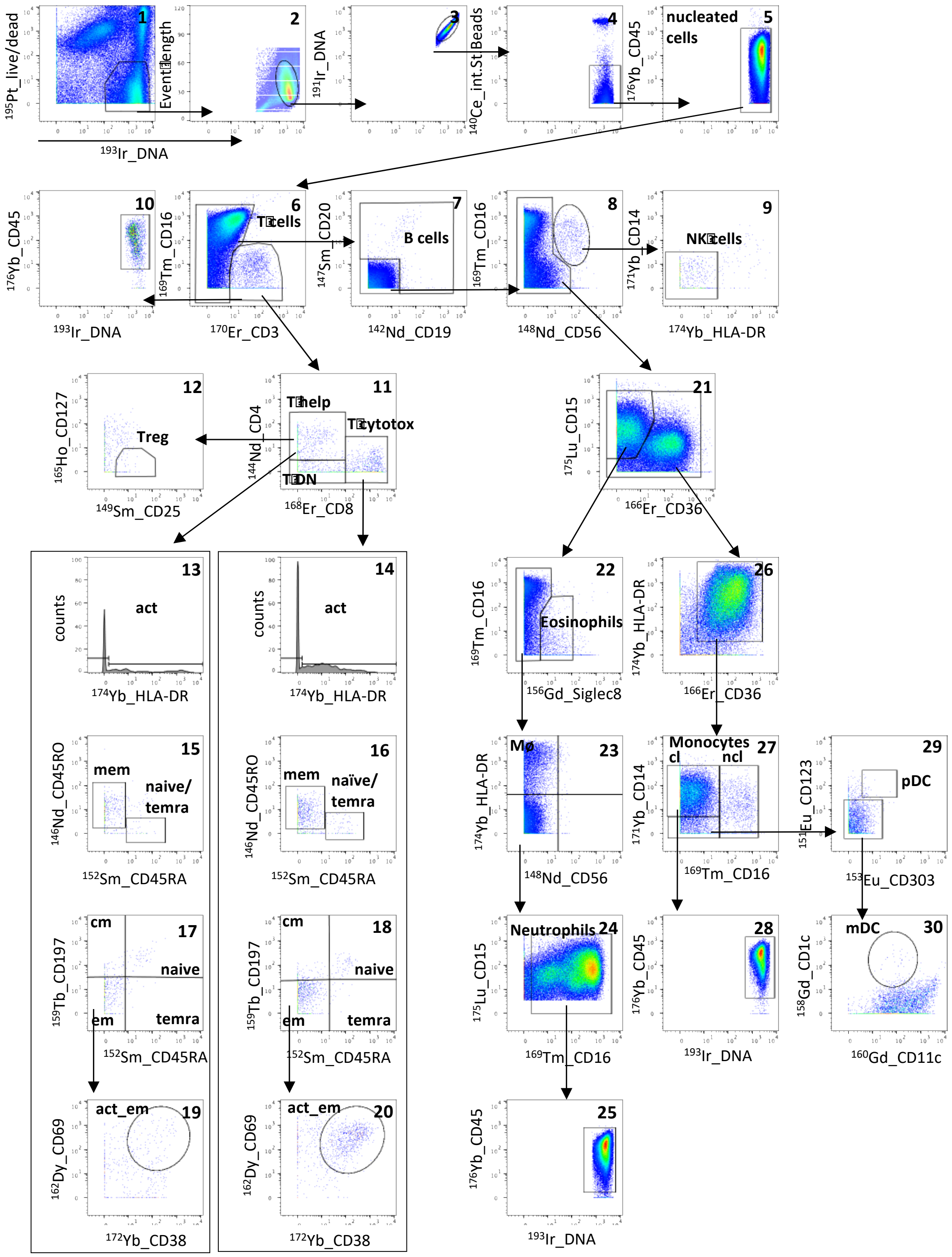

Figure S2: Absolute numbers of live nucleated urine cell counts acquired by mass cytometry

Number of viable urinary cells acquired by mass cytometry according to plot 5 (Figure S1B).

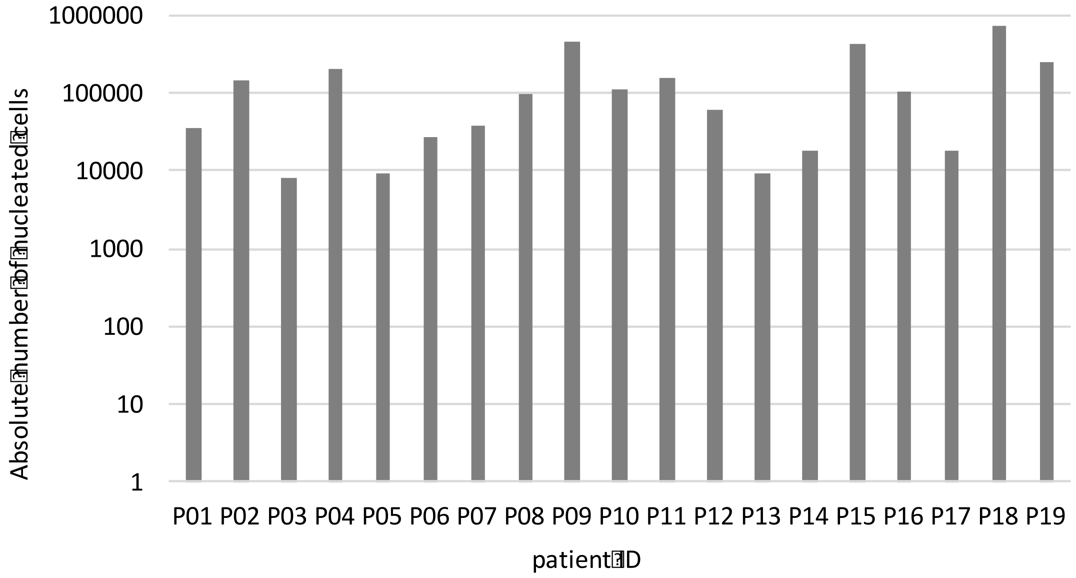

Figure S3: Phenotypic characterization of blood and urinary monocytes/macrophages

Exemplary phenotypic characterization of the Monocyte/Macrophage clusters of blood and urine identified by t-SNE analysis from Figure 3 (P04) for the expression of HLA-DR, CD11c, CD36, CD14 and CD38.

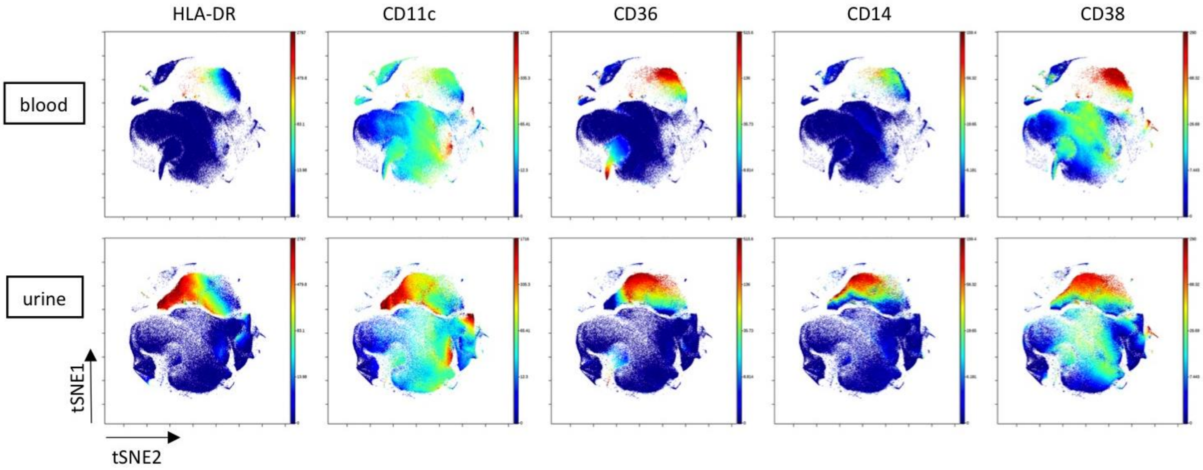

Figure S4: Correlation analysis of T helper cells with proteinuria

A Pearson correlation analysis was performed based on CD4 T cell frequencies and the clinical parameter proteinuria.

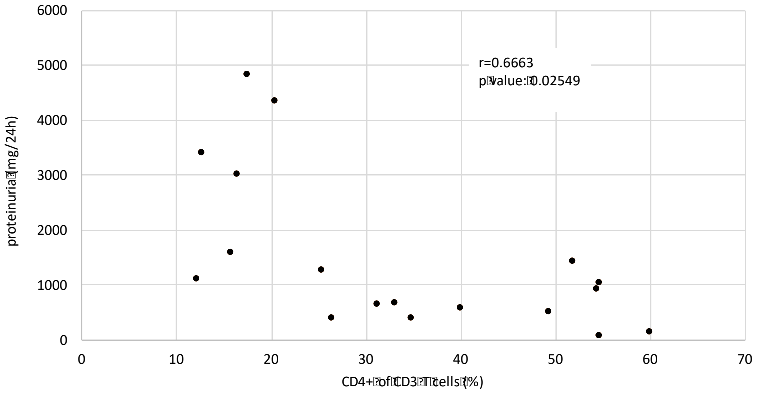

Figure S5: Detection of urinary M2-like macrophages

In two out of six samples (P02 and P04) M2-like macrophages co-expressing $CD163^{+/-}$ and $CD206^{+}$ were detected. The expression of CD163 was significantly decreased in urinary cells if compared to blood monocytes, which did not express CD206.

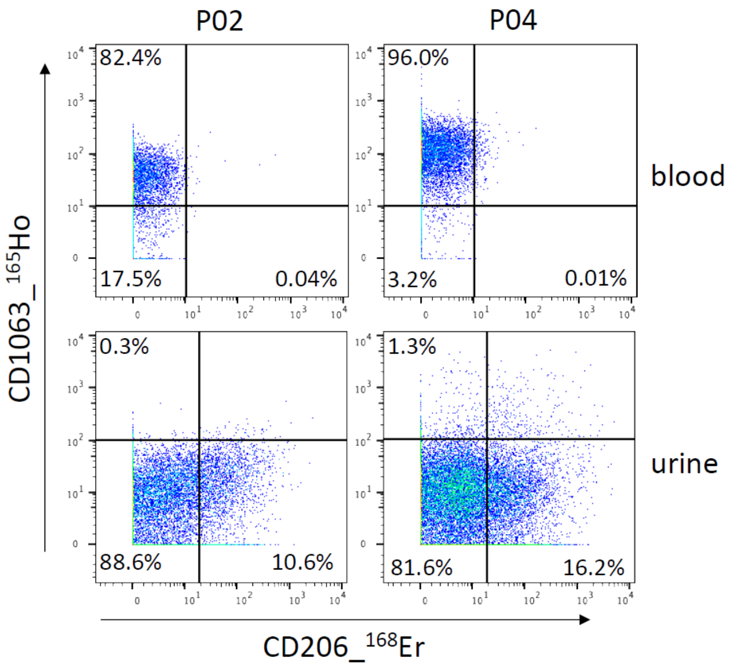

Table S1: List of antibody conjugates and staining reagents used in this study

All pre-conjugated antibodies and MAXPAR labeling kits were purchased from Fluidigm. Unconjugated antibodies were labelled using MAXPAR kits as indicated in the column "Metal-Isotope".

Abbreviations of reference addresses: DRFZ (Deutsches Rheumaforschungszentrum Berlin), Miltenyi (Miltenyi Biotec GmbH), Fluidigm (Fluidigm Corp)

Specificity	Clone	Antibody source	Metal-Isotope
CD45	5B1	Miltenyi	176Yb (MAXPAR)
CD19	Bu-12	DRFZ	142Nd (MAXPAR)
CD4	RPA-T4	BioLegend	144Nd (MAXPAR)
CD45RO	UCHL1	DRFZ	146Nd (MAXPAR)
CD20	2H7	Fluidigm	147Nd
CD56	REA196	Miltenyi	148Nd (MAXPAR)
CD25	2A3	Fluidigm	149Sm
CD169 (Siglec1)	7-239	Miltenyi	150Gd (MAXPAR)
CD123	6H6	Fluidigm	151Eu
CD45RA	4G11	DRFZ	152Sm (MAXPAR)
CD303	201A	Fluidigm	153Eu
CD195 (CCR5)	REA245	Miltenyi	155Gd (MAXPAR)
Siglec 8	7C9	Miltenyi	156Gd (MAXPAR)
CD1c	L161	BioLegend	158Gd (MAXPAR)
CD197 (CCR7)	G0437	Fluidigm	159Tb
CD11c	MJ427G12	Miltenyi	160Gd (MAXPAR)
CD69	FN50	Fluidigm	162Dy
CD127	A019D5	Fluidigm	165Ho
CD36	AC106	Miltenyi	166Er (MAXPAR)
CD27	L128	Fluidigm	167Er
CD8	SK1	Fluidigm	168 Er
CD8	GN11	DRFZ	196Pt (MAXPAR)
CD16	3G8	BioLegend	169Tm (MAXPAR)
CD3	UCHT1	DRFZ	170Er
CD14	TM1	DRFZ	171Yb
CD38	HIT2	Fluidigm	172Yb
HLA-DR	L243	Fluidigm	174Yb
CD15	W6D3	BioLegend	175Lu
cisPlatin	live/dead discrimination	see Materials &Methods	195Pt
intercalator	DNA staining	see Materials &Methods	191Ir/193Ir

Table S2: List of immune cell phenotypes identified in urine

In this table all leukocyte populations were summarized, which could be identified in urine samples by manually gating. Populations marked in red could be not used for hierarchical cluster analysis shown in Figure 4 because absolute number cells was almost >25.

**short name**	**phenotype**	**plot #**
T cells	CD3^{+}CD16^{-} Freq. of nucleated cells	6
B cells	CD3-CD16$^{+/-}$_CD20^{+}CD19^{+}_CD20^{+}CD19^{-}_CD20^{-}CD19^{+} Freq. of nucleated cells	7
NK cells	CD3^{-}CD16$^{+/-}$_CD20^{-}CD19^{-}_CD56^{+}CD16^{+}_CD14^{-}HLA-DR^{-} Freq. of nucleated cells	9
T help	CD3^{+}CD16^{-}_CD4^{+}CD8^{-} Freq. of CD3^{+} T cells	11
T cytotox	CD3^{+}CD16^{-}_CD4^{-}CD8^{+} Freq. of CD3^{+} T cells	11
T DN	CD3^{+}CD16^{-}_CD8^{-}CD4^{-} Freq. of CD3^{+} T cells	11
Treg	CD3^{+}CD16^{-}_CD4^{+}CD8^{-}_CD127lowCD25^{+} Freq. of CD4^{+} T cells	12
act_T help	CD3^{+}CD16^{-}_CD4^{+}CD8^{-}_HLA-DR^{+} Freq. of CD4^{+} T cells	13
act_T cytotox	CD3^{+}CD16^{-}_CD4^{-}CD8^{+}_HLA-DR^{+} Freq. of CD8^{+} T cells	14
naive/temra_T help	CD3^{+}CD16^{-}_CD4^{+}CD8^{-}_CD45RO^{-}CD45RA^{+} Freq. of CD4^{+} T cells	15
mem_T help	CD3^{+}CD16^{-}_CD4^{+}CD8^{-}_CD45RO^{+}CD45RA^{-} Freq. of CD4^{+} T cells	15
naive/temra_T cytotox	CD3^{+}CD16^{-}_CD4^{-}CD8^{+}_CD45RO^{-}CD45RA^{+} Freq. of CD8^{+} T cells	16
mem_T cytotox	CD3^{+}CD16^{-}_CD4^{-}CD8^{+}_CD45RO^{+}CD45RA^{-} Freq. of CD8^{+} T cells	16
cm_T help	CD3^{+}CD16^{-}_CD4^{+}CD8^{-}_CD45RA^{-}CD197^{+} Freq. of CD4^{+} T cells	17
naive_T help	CD3^{+}CD16^{-}_CD4^{+}CD8^{-}_CD45RA^{+}CD197^{+} Freq. of CD4^{+} T cells	17
temra_T help	CD3^{+}CD16^{-}_CD4^{+}CD8^{-}_CD45RA^{+}CD197^{-} Freq. of CD4^{+} T cells	17
em_T help	CD3^{+}CD16^{-}_CD4^{+}CD8^{-}_CD45RA^{-}CD197^{-} Freq. of CD4^{+} T cells	17
cm_T cytotox	CD3^{+}CD16^{-}_CD4^{-}CD8^{+}_CD45RA^{-}CD197^{+} Freq. of CD8^{+} T cells	18
naive_T cytotox	CD3^{+}CD16^{-}_CD4^{-}CD8^{+}_CD45RA^{+}CD197^{+} Freq. of CD8^{+} T cells	18
temra_T cytotox	CD3^{+}CD16^{-}_CD4^{-}CD8^{+}_CD45RA^{+}CD197^{-} Freq. of CD8^{+} T cells	18
em_T cytotox	CD3^{+}CD16^{-}_CD4^{+}CD8^{-}_CD45RA^{-}CD197^{-} Freq. of CD8^{+} T cells	18
act^{-}em_T help	CD3^{+}CD16^{-}_CD4^{+}CD8^{-}_CD45RA^{-}CD197^{-}_CD69^{+}CD38^{+} Freq. of CD4^{+} EM T cells	19
act^{-}em_T cytotox	CD3^{+}CD16^{-}_CD4^{-}CD8^{+}_CD45RA^{-}CD197^{-}_CD69^{+}CD38^{+} Freq. of CD8^{+} EM T cells	20
Eosinophils	CD3^{-}CD16$^{+/-}$_CD20^{-}CD19^{-}_CD16$^{+/-}$CD56^{-}_CD15^{+}CD36^{-}_CD16^{-}Siglec8^{+} Freq. of nucleated cells	22
Mø	CD3^{-}CD16$^{+/-}$_CD20^{-}CD19^{-}_CD16$^{+/-}$CD56^{-}_CD15^{+}CD36^{-}_CD16$^{+/-}$Siglec8^{-}_HLA-DR^{+}CD56^{-} Freq. of nucleated cells	23
Neutrophils	CD3^{-}CD16$^{+/-}$_CD20^{-}CD19^{-}_CD16$^{+/-}$CD56^{-}_CD15^{+}CD36^{-}_CD16$^{+/-}$Siglec8^{-}_HLA-DR^{-}CD56^{-}_CD15$^{+/-}$CD16^{+} Freq. of nucleated cells	24
cl_Monocytes	CD3^{-}CD16$^{+/-}$_CD20^{-}CD19^{-}_CD16$^{+/-}$CD56^{-}_CD15$^{+/-}$CD36^{+}_HLA-DR^{+}CD36^{+}_CD14^{+}CD16^{-} Freq. of parent	27
ncl_Monocytes	CD3^{-}CD16$^{+/-}$_CD20^{-}CD19^{-}_CD16$^{+/-}$CD56^{-}_CD15$^{+/-}$CD36^{+}_HLA-DR^{+}CD36^{+}_CD14$^{+/-}$CD16^{+} Freq. of parent	27
pDC	CD3^{-}CD16$^{+/-}$_CD20^{-}CD19^{-}_CD16$^{+/-}$CD56^{-}_CD15$^{+/-}$CD36^{+}_HLA-DR^{+}CD36^{+}_CD14^{-}CD16^{-}_CD123^{+}CD303^{+} Freq. of nucleated cells	29
mDC	CD3^{-}CD16$^{+/-}$_CD20^{-}CD19^{-}_CD16$^{+/-}$CD56^{-}_CD15$^{+/-}$CD36^{+}_HLA-DR^{+}CD36^{+}_CD14^{-}CD16^{-}_CD123^{-}CD303^{-}_CD1c^{+}CD11c^{+} Freq. of nucleated cells	30

cytotox: cytotoxic; DN: double negative; act: activated; mem: memory; cm: central memory; em: effector memory; temra: terminally differentiated effector cells; Mø: macrophages; cl: classical; ncl: non-classical; Freq: Frequency: pDC: plasmacytoid dendritic cells; mDC: myeloid dendritic cells
